# A *LexAop* > *UAS* > *QUAS* trimeric plasmid to generate inducible and interconvertible *Drosophila* overexpression transgenes

**DOI:** 10.1038/s41598-022-07852-7

**Published:** 2022-03-09

**Authors:** Franz Wendler, Sangbin Park, Claire Hill, Alessia Galasso, Kathleen R. Chang, Iman Awan, Yulia Sudarikova, Mar Bustamante-Sequeiros, Sichen Liu, Ethan Y-H. Sung, Gabrielle Aisa-Bonoko, Seung K. Kim, Luis A. Baena-Lopez

**Affiliations:** 1 Sir William Dunn School of Pathology, University of Oxford, South Parks Road, Oxford, UK; 2 Department of Developmental Biology, Stanford University School of Medicine, Stanford, CA USA; 3 Department of Medicine, and Stanford Diabetes Research Center, Stanford University School of Medicine, Stanford, CA USA

**Keywords:** Gene expression, Genetic vectors

## Abstract

The existence of three independent binary systems for conditional gene expression (*Gal4/UAS; LexA/LexAop; QF/QUAS*) has greatly expanded versatile genetic analyses in the *Drosophila melanogaster*; however, the experimental application of these tools is limited by the need to generate multiple collections of noninterchangeable transgenic fly strains for each inducible gene expression system. To address this practical limitation, we developed a modular vector that contains the regulatory elements from all three binary systems, enabling Gal4-, LexA- or QF-dependent expression of transgenes. Our methods also incorporate DNA elements that facilitate independent site-specific recombination and elimination of regulatory *UAS*, *LexAop* or *QUAS* modules with spatial and temporal control, thus offering unprecedented possibilities and logistical advantages for in vivo genetic modulation and efficient interconversion of overexpression transgenic fly lines.

## Introduction

The inducible *Gal4/UAS* gene expression system revolutionized genetic experimentation in fruit flies^1^. This binary genetic tool facilitates gene expression in larval and adult fly tissues with cellular specificity and temporal control^1^. The *Gal4/UAS* system relies on the production of the yeast Gal4 transcription factor from gene-specific regulatory enhancers and adjacent promoters^1^. The Gal4 protein can bind to cognate DNA upstream activating sequences (*UAS*), thereby inducing transcription of any DNA sequence of interest inserted downstream of *UAS*^1^. The *Gal4/UAS* system has been instrumental in deciphering many biological processes and has inspired the development of analogous conditional binary gene expression tools such as the *LexA/LexAop* and *QF/QUAS* systems^2,3^. The use of these bipartite gene expression tools, singly or in combination, has greatly broadened conditional genetics in fruit flies^4^. However, each of these gene expression tools requires the generation of independent transgenic fly lines, limiting experimental interchangeability and combinatorial usage, as well as generating cost inefficiencies related to the production and maintenance of multiple transgene collections.

Beyond bipartite overexpression systems, conditional gene expression has successfully been achieved in *Drosophila* by using three sequence-specific recombination methods. The most popular is the *Flippase/FRT* (*Flp/FRT*) system^5^, which capitalizes on the Flp-dependent DNA recombinase activity of Flp-recognition sites (*FRT* sequences)^5^. Recently, directed mutagenesis of the ‘wild type’ Flp enzyme (Flp^WT^, hereafter) and the *FRT*-recognition sites generated the *mFlp5/mFRT71* system^6^. Another alternative, the *Cre/LoxP* system, relies on Cre-mediated recombination of *LoxP*-recognition motifs^7^. No cross-reactivity has been reported between these three recombination systems^6,8^ and therefore they can be used in parallel.

We describe a modular vector that allows gene expression, singly or in combinations, through the three most popular bipartite activation systems. It also includes different recombination sites that facilitate conditional termination of gene expression with spatial and temporal precision in both somatic and germline cells. Together, these features open new experimental opportunities for *Drosophila* researchers and the generation of interconvertible fly overexpression repositories with important logistic savings.

## Results

### Modular transcriptional activation repeats facilitate flexible gene expression in *Drosophila* through any of the bipartite activation systems

**Figure 1:**
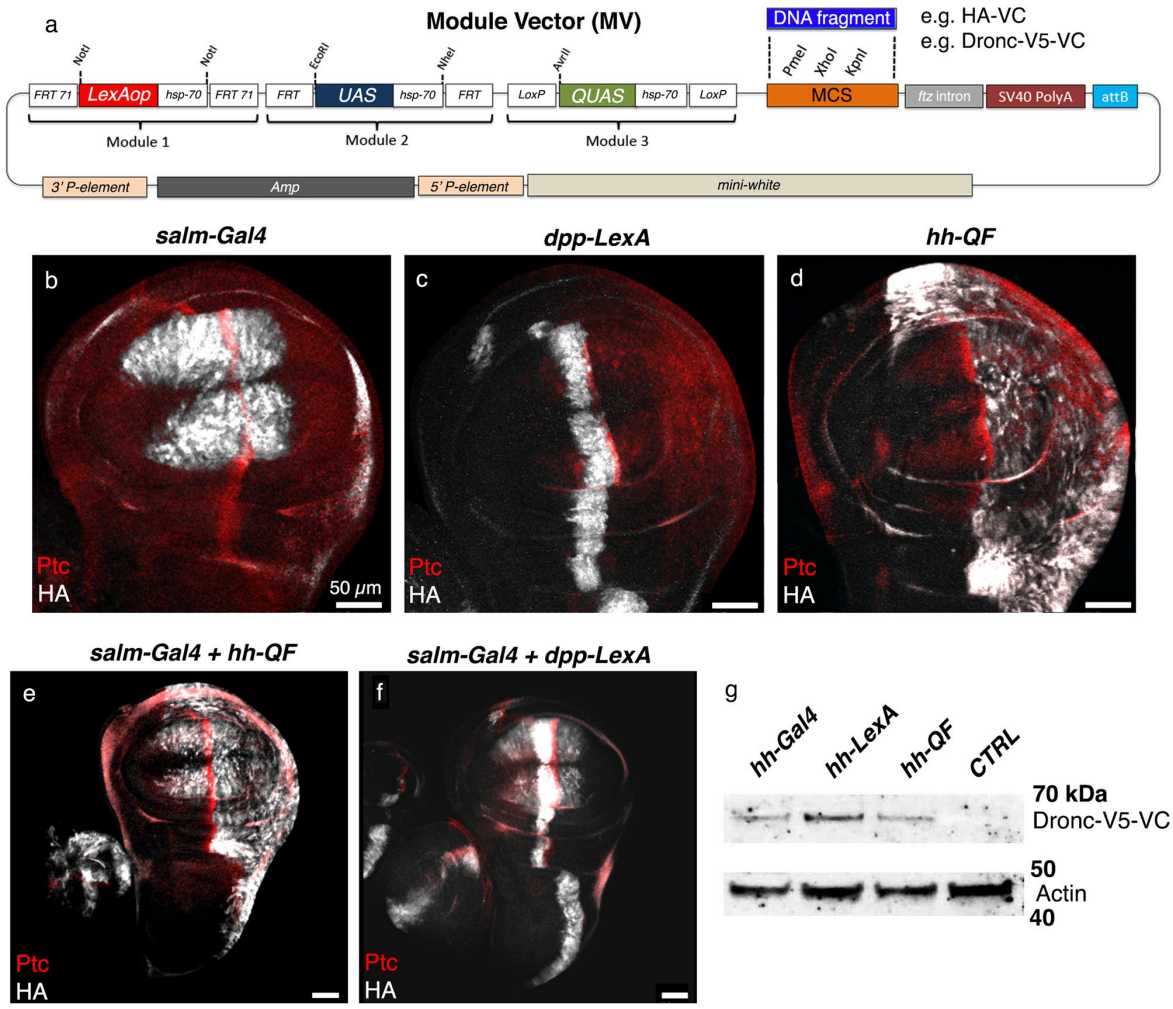
Features of the new Modular Vector (*MV*). (**a**) Schematic showing the different features incorporated into the modular vector (*MV*). (**b–d**) Expression of HA-VC (anti-HA immunostaining, gray) within discrete cellular domains of the wing disc under the regulation of different transcriptional activators. (**e–f**) Concomitant expression of HA-VC in several cellular domains of the wing disc under the regulation of two different transcriptional activators. Patch expression (anti-Ptc immunostaining, red) labels the confrontation between anterior and posterior cells of the wing disc. Scale bars represent 50 μm in the entire figure. (**g**) Western Blot shows the expression of the full-length Dronc-V5-VC construct induced by *hh-Gal4, hh-LexA*, and *hh-QF*. The control empty (CTRL) sample was obtained from flies *hh-lexA* without the *MV-Dronc-V5-VC* transgene. The expected sizes of Dronc-V5-VC and Actin are approximately 62 and 42 kDa, respectively.

To circumvent the shortcomings of *Drosophila* binary gene expression systems, we developed a modular vector (*MV*, hereafter) that incorporates transcriptional binding sites for the three most popular bipartite systems (5 × *LexAop* / 5 × *UAS* / 5 × *QUAS*), upstream of a multicloning site (Fig. 1a, Supplementary Text). Each pentameric activation sequence was followed by a minimal *hsp-70* promoter^9^ to ensure robust gene expression. In addition, after the multicloning site, *MV* contains a *fushi tarazu (ftz)* intron followed by a *SV40 poly(A)* tail that ensure transcription termination and mRNA stability^10^. Downstream of the *SV40 poly(A)*, *MV* also incorporates an *attB* recombination site to facilitate the generation of transgenic flies using PhiC31-mediated integration^11^. This design was predicted to enable robust  in vivo expression of cDNA under the regulation of different transactivators, and ease generation of transgenic flies containing *MV-*based constructs.

To demonstrate the flexible function of the *MV* and facilitate future experiments in our laboratory, we generated a transgenic fly line encoding a synthetic protein containing the small peptide HA fused to a fragment of a split Venus fluorescent protein (VC fragment)^12^, which can be recognised using an antibody against GFP (Methods). We subsequently intercrossed the new *MV*-*HA-VC* flies with other fly strains expressing different transcriptional activators (*decapentaplegic-LexA, dpp-LexA*^13^; *spalt major-Gal4*, *salm-Gal4*^14^; or *hedgehog-QF*, *hh-QF*^15^). Each of these intercrosses generated F1 larval progeny and restricted HA-VC expression within *Gal4-, LexA-* and *QF-*transcribing cells (Fig. 1b–d). Furthermore, the concomitant use of two transcriptional drivers (*dpp-LexA/hh-QF*; *dpp-LexA/salm-Gal4*) induced the simultaneous expression of HA-VC within their cellular domains (Fig. 1e,f). Confirming the expression of our full-length HA-VC protein, immunostaining for HA and VC (using anti-GFP antibody) colocalized in all of the cells overexpressing the construct (Supplementary Fig. 1b). Similar results were obtained expressing a form of the initiator caspase Dronc tagged with V5 and VC peptides at the C-terminus (Supplementary Fig. 1c–e). In this instance, we confirmed the full-length expression of the Dronc-V5-VC construct by Western blot (the expected size of Dronc-V5-VC is ~ 62 kDa; Fig. 1g, Supplementary Fig. 2a). These results demonstrated that our method allows the expression of full-length cDNAs under either simultaneous or combinatorial control of different bipartite gene expression systems. We speculate that enhanced spatial and temporal precision will be achievable by incorporating Gal80 or QS, the respective repressors of Gal4/LexA or QF^4^.

Next, we evaluated whether comparable levels of overexpression were induced by each transcriptional activation module and they did not influence each other due to their positional location within *MV*. To this end, we assessed the immunofluorescence intensity of *Dronc-V5-VC* induced by *hh-Gal4, hh-LexA*, and *hh-QF* along the dorso/ventral boundary of the posterior compartment of wing discs (Supplementary Fig. 2b–c). Paradoxically, these experiments revealed that *hh-Gal4* and *hh-LexA* induced comparable levels of *Dronc-V5-VC* and, in turn, significantly higher to that obtained with *hh-QF* (Supplementary Fig. 2d). These results ruled out additive effects due to the presence of several *hsp-70* repeats. They also suggested that each regulatory module in *MV* acts independently and only uses the closest *hsp-70* promoter. Together, *MV* features can streamline genetic configurations for investigating gene function in cells with different origins and for studying interorgan cell communication (e.g., intercellular communication between neurons and muscle cells).

Lastly, we examined the ability of *MV*-based transgenes to generate loss-of-function phenotypes expressing short-hairpin RNA interference constructs. To this end, we cloned in the multicloning site of *MV* short inverted repeats (https://flybase.org/reports/FBtp0065104) that were successfully used to target the expression of *wingless (wg)*. Specifically, a *UAS* transgenic line expressing this construct was shown to compromise *wg* expression in wing imaginal discs under the regulation of *patch-Gal4*^16^. However, our *MV*-*wg*-*shRNA* transgene failed to induce noticeable Wingless downregulation or *wg*-mutant phenotypes using several transcriptional drivers (e.g., *engrailed-Gal4, hh-QF* and *dpp-LexA*). These preliminary experiments do not fully discard the potential of *MV* to support RNA interference but evidence important limitations, and therefore further optimization of *MV* is likely needed for this particular application. One potential modification could be the incorporation of a higher number of transcriptional binding sites. *MV* currently contains five transcriptional activation sequences for each bipartite system and the best performance for RNAi applications has been achieved using at least ten binding repeats^10,17,18^. Despite the aforementioned shortcomings, *MV* retains its usefulness for a wide range of biological applications requiring gene overexpression.

### Conditional termination of gene overexpression in somatic cells aided by specific recombination sites

**Figure 2:**
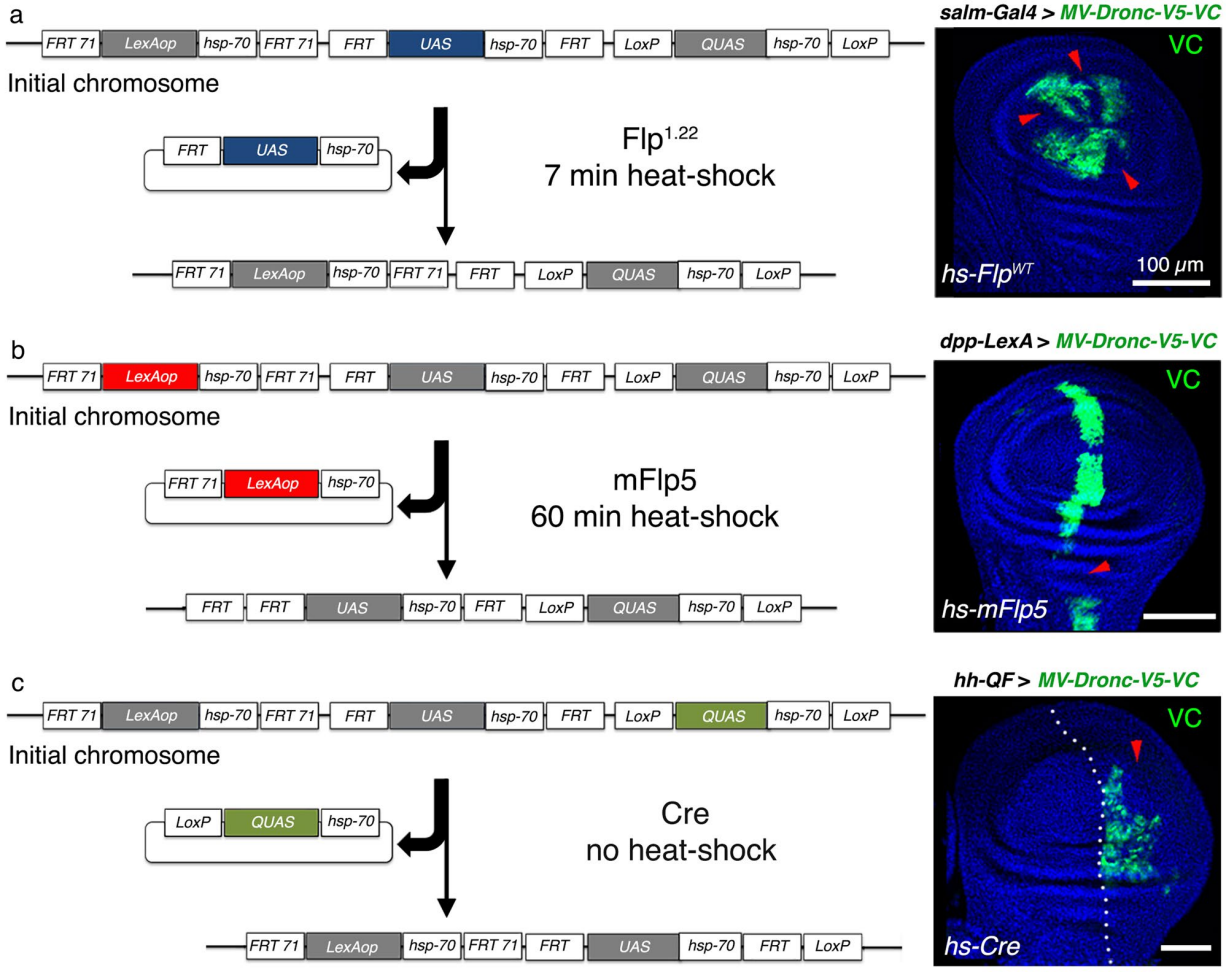
Generation of genetic mosaics in somatic tissues upon genomic elimination of specific gene regulatory elements included in the *MV*. (**a–c**) Clonal elimination from the genome of 5X *UAS* (**a**), 5X *LexAop* (**b**), and 5X *QUAS* (**c**) binding sites upon random exposure to Flp^1.22^ (**a**), mFlp5 (**b**) and Cre (**c**); anti-GFP was used to detect the VC tag of the Dronc-V5-VC construct (green) and DAPI (blue) labels the DNA; red arrowheads indicate the lack of expression of Dronc-V5-VC in the expression domain of the different drivers (*Gal4, LexA,* and *QF*). Compare the expression of Dronc-V5-VC (green) in this figure and Supplementary Fig. 1. Note that the *hs-Cre* transgene is basally expressed in random cells without heat shock induction. Scale bars represent 100 μm in the entire figure.

Different recombination systems without cross-reactivity (*Flp/FRT*, *mFlp5/mFRT71*, and *Cre/loxP*) have been used for conditional gene expression in *Drosophila*^6,8^. To facilitate the regulated termination of gene overexpression using *MV*, we flanked each pentameric regulatory module by specific pairs of recombination motifs (Fig. 1a). The *UAS* repeats were encompassed by *FRT* recombination motifs (Flp sensitive), whereas the *QUAS* and *LexAop* repeats were enclosed between *loxP* (Cre sensitive) and *mFRT71* sites (mFlp5 sensitive) (Fig. 2a), respectively. Our subsequent functional analyses indicated that each type of gene regulatory element was specifically excised from the genome in somatic cells upon expression of the cognate recombinase (Flp^1.22^, Cre or mFlp5) (Fig. 2a–c; Methods). This process was readily visualized by the appearance of genetic mosaics formed by somatic cells failing to express the protein of interest (VC-negative cells without anti-GFP immunostaining indicated by red arrowheads in the right panels of Fig. 2a–c). Next, we assessed whether prolonged expression of the various recombinases would suppress the overexpression of the transgene of interest in most of the cells forming specific cellular territories. Whereas relatively short exposure to Flp^1.22^ and Cre (30 min.) almost fully eliminated the overexpression of Dronc-V5-VC induced by *salm-Gal4* and *hh-QF* drivers, 90 min. of mFlp5-exposure were not sufficient to do so under the regulation of *dpp-LexA* (Supplementary Fig. 3). These results indicated that excision of *UAS-* and *QUAS*-repeats is feasible in large cellular domains by prolonging the heat-shock treatment; however, similar results are difficult to achieve using the mFlp5 likely due to a reduced recombination efficiency.

### Interconvertible generation of *Drosophila* strains from a unique founder line

**Figure 3:**
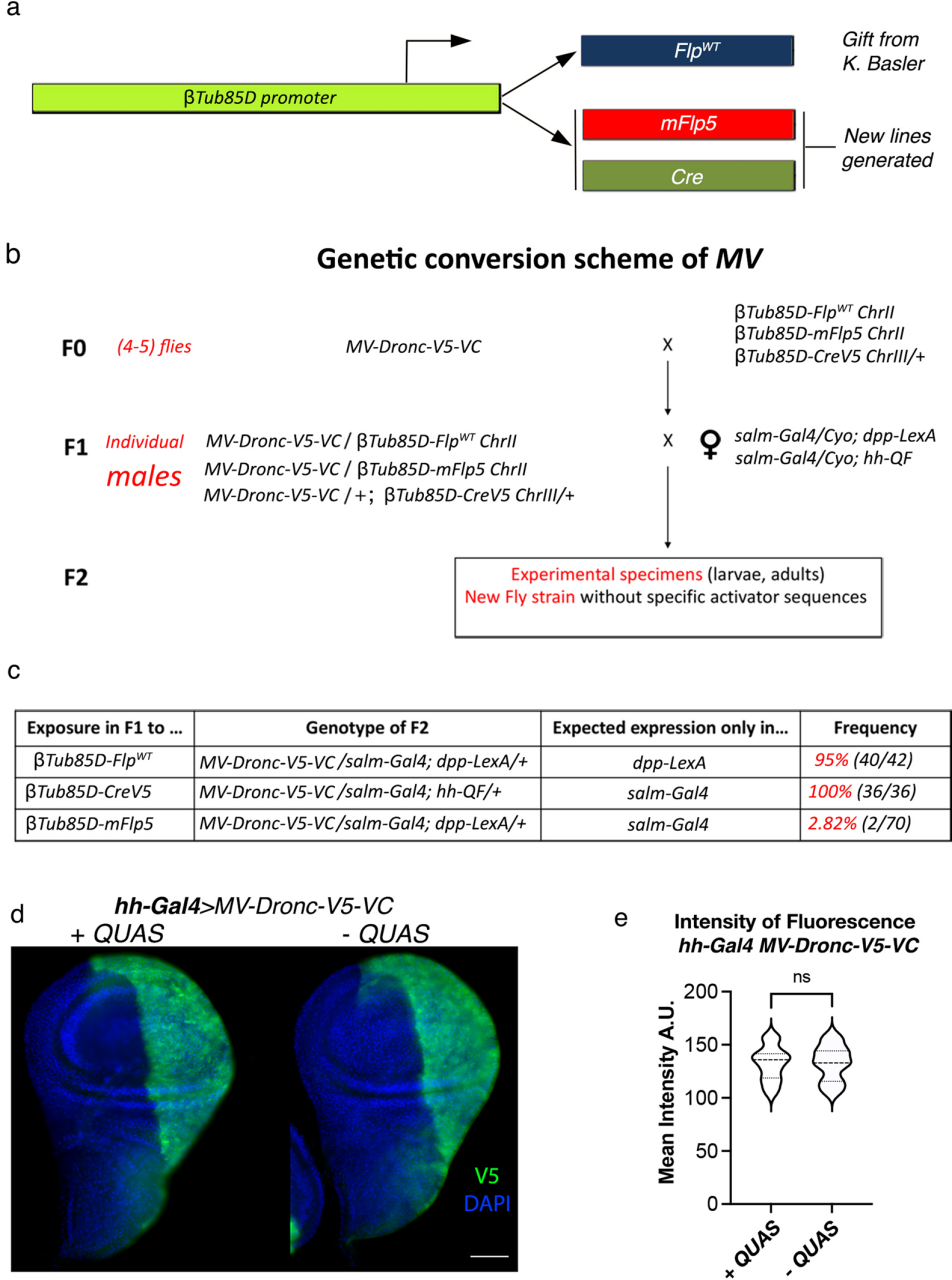
Genetic strategies to permanently eliminate specific activating sequences in the germline. (**a**) Schematic showing the configuration of fly strains previously available (*βTub85D- Flp*^*WT*^*)* or newly generated (*βTub85D-mFlp5 and βTub85D-Cre)* to express different recombinases under the regulation of the *βTub85D* promoter. (**b**) Genetic scheme of intercrosses to permanently eliminate regulatory components of *MV* constructs in the male germline; this feature can be used to create new transgenic flies containing only a subset of activating binding sites. (**c**) Frequency of germline excision of each gene regulatory element upon exposure to different recombinases. (**d**) Representative of images wing imaginal discs expressing Dronc-V5-VC (anti V5 in green) under the regulation of *hh-Gal4* in the posterior compartment using either a fly strain with (+ *QUAS*) or without the *QUAS* (-*QUAS*) repeats. DAPI (blue) labels DNA. Scale bar: 50 μm. e) Mean fluorescence intensity (A.U.) of V5 immunostaining in the P compartment of wing discs of the genotypes indicated in d. Statistical significance was determined using an unpaired Student’s t-test; not significant, n.s. number of discs + *QUAS* n = 20 and – *QUAS* n = 16; N = 2. Full genotype descriptions of the figure can be found in Methods.

Next, we assessed whether gene activation sequences in our modular activation construct could be permanently excised in the germline, thus facilitating the generation of new strains with a subset of gene activation sequences derived from a single founder line. To this end, we intercrossed the *MV*-*Dronc-V5-VC* founder strain with flies expressing Flp^WT^ under the regulation of a *βTubulin 85D*-promoter (*βTub85D-Flp*^*WT*^*,* hereafter; this fly strain was kindly provided by Professor Konrad Basler) (Fig. 3a). The activity of *βTubulin 85D*-promoter is highly restricted to the male germline^19,20^, and therefore, it was expected to induce efficient recombination between *FRT* sites within male gametes. After the initial set of crosses (please see diagram of intercrosses in Fig. 3), we retained the F1 male progeny containing both transgenes (*MV*-*Dronc-V5-VC* and *βTub85D-Flp*^*WT*^). These males were next intercrossed with females that concomitantly expressed *salm*-*Gal4* and *dpp*-*LexA* transcriptional activators (Fig. 3b). Ninety-five percent of the progeny did not express the Dronc-V5-VC construct in the *salm-Gal4* domain (40/42 wing discs from 26 larvae collected from 5 independent crosses) but retained activation in the *dpp-LexA* territory (Fig. 3c). These results demonstrated that most of the male gametes expressing Flp^WT^ permanently excised *UAS* regulatory repeats without compromising the *LexA*-binding sites. Comparable results were obtained when Cre was expressed under the regulation of the *βTub85D* promoter (*βTub85D-Cre)* (Fig. 3b, c). Unlike the  previously described results, the  presence of mFlp5 in the male germline (*βTub85D-mFlp5*) only affected the expression of Dronc-V5-VC in approximately 2.85% of analysed larvae (2/70 wing discs from 35 larvae). These results are likely due to the lower recombination efficiency of mFlp5. Despite this potential limitation, our findings confirm the suitability of *MV* to efficiently derive new fly strains, with specific subsets of transcriptional binding sites, from a single founder line through a simple scheme of intercrosses (Fig. 3b). Moreover, the efficient germline excision of specific regulatory elements allows the use of novel *MV* fly strains with previously created lines in public repositories for combinatorial gene overexpression.

Finally, we determined whether the permanent elimination of specific activation sequences had an influence on the levels of overexpression induced by the others. To this end we assessed whether the elimination of the *QUAS-* repeats affected the overexpression levels of Dronc-V5-VC induced by *hh-Gal4*. However, we did not observe significant differences between the *MV* transgene with or without the *QUAS*-sequences (Fig. 3d–e). These results confirmed the independent behaviour of each transcriptional activation module.

## Discussion

We have generated a next-generation plasmid (*MV*) that provides enhanced versatility to conduct targeted genetics in *Drosophila*. Our results indicate that our method is able to induce gene overexpression through any of the three most popular *Drosophila* bipartite systems, either singly or in combination. In addition, gene overexpression can be suppressed with high precision by taking advantage of specific recombination sites flanking the transcriptional activation sequences. These features multiply the experimental possibilities to manipulate gene expression with spatial and temporal precision in several cell populations and could be highly useful to investigate intercellular communication between cells with a different genetic configuration. This could be also instrumental to model aspects of specific diseases in *Drosophila* involving several cell types at the same time. A shortcoming of our method seems to be its limited compatibility to support RNA interference, possibly due to the low number of gene activation sequences (only 5 repeats). However, this does not compromise the wide range of gene overepexresion applications offered by *MV*. Beyond the advantages of manipulating gene expression in somatic cells, we have shown the suitability of *MV* to efficiently create new fly strains that retain only a subset of activation sequences (Fig. 3). Importantly, these new lines can be derived from a unique founder strain after completing a simple genetic scheme of intercrosses. This could substantially reduce the logistic costs and personnel efforts to develop interconvertible repositories of fly overexpression transgenes.

## Material and methods

### Molecular cloning

All PCRs were performed with Q5 High-Fidelity polymerase from New England Biolabs (NEB, M0492 L). Standard subcloning protocols were used to generate all the DNA plasmids (see details below). Genomic DNA was extracted via standard protocols^21^ and used as a template to amplify different DNA fragments. Transgenic flies harbouring the new transgenes were obtained by *attP/attB* PhiC31-mediated integration (see the specific details below). Some of the transgenes were generated by Bestgene Inc., while others were generated in house by Sangbin Park. The fly strains generated will be deposited at the Bloomington Stock Centre. While resources are transferred to Bloomington, reagents will be provided upon reasonable request.

#### Generation of the Module Vector (MV)

The different modules included in the vector were first designed in silico using SnapGene software. Each module contains 5 repeats of each upstream regulatory sequence type, followed by an *hsp-70* minimal promoter. Each module was also flanked by specific recombination sites (see diagram Fig. 1). This fragment was synthesized as a large DNA fragment by GENEWIZZ and subcloned into the PUC57 vector. The modular construct was then extracted from PUC57 as a SpeI-KpnI fragment and subcloned into a *UASt-attB-mini-white*^11^ vector previously digested with NheI-KpnI. PmeI, XhoI, and KpnI are potential unique restriction sites available for conventional cloning downstream of the gene regulatory elements (see diagram Fig. 1). Sequence of the plasmid is provided as Supplementary Text information.

#### Generation of *βTub85D-mFlp5* and *βTub85D-CreV5* transgenic flies

We extracted genomic DNA and amplified a *βTub85D* promoter via PCR using the following primers:

Forward primer *βTub85D*-promoter:

*5’ ttattatccctaggcagctgtggactcctcattgtagg 3’*

Reverse primer *βTub85D*-promoter*:*

*5’ aaatttaatctgcaggcggccgcgaattcaagcttcgcccctttttcacaccg 3’*

Convenient restriction sites for cloning were placed at the 5’ (AvrII) and 3’ (NotI and EcoRI) ends of the PCR product. We then digested a *UASt-attB-mini-white* vector with NheI and EcoRI, thus replacing the *UAS* repeats with the *βTub85D*-promoter but keeping the rest of the plasmid backbone. PCR was digested with AvrII and EcoRI and ligated with the aforementioned backbone, creating an intermediate plasmid (*βTub85D-attB-mini-white* plasmid) suitable to subclone the cDNA of *mFlp5* and *CreV5*. *mFlp5* cDNA was amplified via PCR from a plasmid vector kindly provided by Iris Salecker using the following primers:

Forward primer *mFlp5*:

*5’* *attacagttGCGGCCGCatgccacaatttgatatattatgtaaaacacc 3’*

Reverse primer *mFlp5:*

*5’ AAtATAaaggcctTctagattatatgcgtctatttatgtagg 3’*

The NotI and StuI restriction sites were conveniently placed in the PCR product of *mFlp5* to facilitate cloning in the *βTub85D*-attB plasmid. Before ligation, the *βTub85D-attB* plasmid and the *mFlp5* PCR product were digested with NotI and StuI. This construct was inserted in the *attP* site of the Bloomington Stock Number 9740.

To generate the *βTub85D*-*CreV5*-*attB-mini-white* vector, we replaced the *UAS* repeats included in *UASt-Crev5-attB-mini-white* plasmids previously generated in the laboratory with the Beta2-Tubulin minimal promoter. To that end and prior to ligation, we digested the *UASt-CreV5-attB-mini-white* plasmid and the *βTub85D-*promoter PCR product with NheI-EcoRI. Note that Cre was tagged with the V5 epitope. This construct was inserted in the *attP* site of the Bloomington Stock Number 9738 to generate the corresponding transgenic line.

##### *MV-HA-VC*

The VC fragment corresponding to the split Venus GFP^12^ was amplified by PCR with the primers indicated below using as template the Addgene plasmid number 22011. The HA epitope was incorporated in the N-terminus within the forward primer. Primers also contained suitable restriction sites to facilitate subcloning. The PCR product was first subcloned as a PmeI-XhoI fragment in a customised *Actin5C-SV40-polyA-attB-mini-white* plasmid^22^. Finally, the HA-VC fragment with the SV40 polyA was subcloned in the *MV* vector using the PmeI-SpeI restriction sites. Sequence of the plasmid will be provided upon request until the vector is deposited in a public repository. This construct was inserted in the *attP* site of the Bloomington Stock Number 9752 to generate the corresponding transgenic line.

Forward primer HA-VC:

*5’ttaggcggtttaaacgcggccgcgccaccgacgtcatgtacccatacgatgttccagattacgctggggccgcgg*

*ccggggacaagcagaagaacg 3’*

Reverse primer VC*:*

*5’attatagagctcgaggtaccctactattacttgtacagctcgtccatgccgagagtgatccc 3’*

#### *MV-Dronc-V5-VC*

The Dronc-V5-VC fragment was synthesized by Twist Bioscience. Wild-type cDNA of Dronc was fused to the V5 and VC peptides at the C-terminus. The constructs were subcloned in the *MV* vector using PmeI-KpnI restriction sites. Sequence of the plasmid will be provided upon request until the vector is deposited in a public repository. This construct was inserted in the *attP* site of the Bloomington Stock Number 9752 to generate the corresponding transgenic line.

#### *MV-wg-RNAi*

The construct was built using the primers indicated below. The primers incorporate the inverted repeats for targeting the gene *wingless* previously described in the *Drosophila* TRiP collection (HMS00844) (https://fgr.hms.harvard.edu/fly in vivo-rnai). The primers were synthesized and subsequently annealed as follows. We prepare 50 µl of a solution containing both oligos (final concertation 20 µM) in buffer 2.1 10X (New England Biolabs) and the corresponding water volume. This mix was heated for 5 min in a thermoblock and left at room temperature during 2 h prior ligation in the *MV* vector opened with PmeI-KpnI restriction sites. This construct was inserted in the *attP* site of the Bloomington Stock Number 9753 to generate the corresponding transgenic line.

Forward primer:

*5’ aaaccagttagctcgatatgaatataatatagttatattcaagcatatattatattcatatcgagctagcggtac 3’*

Reverse primer*:*

*5’ cgctagctcgatatgaatataatatatgcttgaatataactatattatattcatatcgagctaactggttt 3’*

### Fly Husbandry and full description of genotypes

All fly strains used are described at www.flybase.bio.indiana.edu unless otherwise indicated. Primary *Drosophila* strains and experiments were routinely maintained on Oxford fly food at 25 °C.

### Full genotype description

#### Figure 1

1b: *salm-Gal4*/*MV*-*HA-VC.*

1c: *MV*-*HA-VC/* + *; dpp-LexA*/ + 

1d: *MV*-*HA-VC/* + *; hh-QF*/ + 

1e: *salm*-Gal4/*MV*-*HA-VC; hh-QF*/ + 

1f.: *salm-Gal4*/*MV*-*HA-VC; dpp-LexA*/ + 

1 g: *MV*-*Dronc-V5-VC*/Cyo; *hh-Gal4*/ + 

*MV-Dronc-V5-VC*/Cyo; *hh-LexA*/ + 

*MV-Dronc-V5-VC*/Cyo; *hh-QF*/ + 

CTRL: + /Cyo; *hh-LexA*/ + 

#### Figure 2

2a: *hs*-*Flp*^*1*.22^; *salm-Gal4*/*MV*-*Dronc-V5-VC*

2b: *MV*-*Dronc-V5-VC/* + *; dpp-LexA*/*hs-mFlp5*

2c: *MV*-*Dronc-V5-VC/hs-Cre; hh-QF*/ + 

#### Figure 3

3b-c: The genotypes are indicated in the figure

3d and 3e:

*MV-Dronc-V5-VC*/Cyo; *hh-Gal4*/+ using a *MV-Dronc-V5-VC* transgene with (+ *QUAS*) or without (- *QUAS*) repeats. The *QUAS* repeats were permanently removed in the germline following the protocol described in Figure 3.

#### Supplementary Fig. 1

1b: *salm-Gal4*/*MV*-*HA-VC; hh-QF*/ + 

1d: *salm-Gal4*/*MV*-*Dronc-V5-VC; dpp-LexA*/ + 

1e: *salm-Gal4*/*MV*-*Dronc-V5-VC; hh-QF*/ + 

#### Supplementary Fig. 2

2a: Genotypes are the same than in the Fig. 1g.

2b and 2e:

MV-*Dronc-V5-VC*/Cyo; *hh-Gal4*/ + 

MV-*Dronc-V5-VC*/Cyo; *hh-LexA*/ + 

MV-*Dronc-V5-VC*/Cyo; *hh-QF*/ + 

#### Supplementary Fig. 3

3a: *hs-Flp*^*1*.22^; *salm-Gal4*/*MV*-*Dronc-V5-VC.*

3b: *MV*-*Dronc-V5-VC/hs-Cre; hh-QF*/ + 

3c: *MV*-*Dronc-V5-VC/* + *; dpp-LexA*/*hs-mFlp5.*

### Immunohistochemistry

Third instar larvae were dissected on ice-cold PBS. The larvae were then fixed and immunostained following standard protocols (fixing solution 4% paraformaldehyde diluted in PBS 1X; washing solution 0.3% Triton X-100 diluted in PBS 1X). The primary antibodies used in our experiments were anti-GFP (goat, 1:400; Abcam, ab6673), anti-Ptc (1:200, Hybridoma Bank, Apa1), anti-V5 (mouse 1:200, Thermofisher R960-25) and anti-HA (rabbit 1:500, Cell Signaling 3724). We diluted the secondary antibodies in a solution 0.3% Triton X-100 diluted in PBS 1X to detect the primary antibodies: anti-goat Alexa 488 (A1105), anti-mouse Alexa 555 (A31570), and anti-rabbit Alexa 647 (A31573). All of the secondary antibodies were from Life Technologies and were used at a standard concentration of 1:200. DAPI was added to the solution with secondary antibodies to label the DNA (1:1000; Thermo Scientific 62248). After incubation for 2 h with the secondary antibodies, samples were washed 3 times in a solution 0.3% Triton X-100 diluted in PBS 1X for 5 min and mounted in Vectashield.

### Western Blot

20 wing imaginal discs from each genotype were collected in 20 ml PBS 1X and snap-frozen at -80C, thawed and macerated in SDS loading buffer (Invitrogen; NP008) in a volume of 40 µl, and cleared at 20.000 g for 5 min. Lysates were separated on a 12% precast SDS-PAGE (Invitrogen, NP0341 BOX). Dronc-V5-VC expression was detected in the Western blot using a goat anti-GFP antibody (1:2500; Abcam, ab6673) and beta-actin (DSHB 1:500).

### Imaging of wing discs

Confocal imaging of wing imaginal discs was performed using the Olympus Fluoview FV1200 and the associated software. Fifty-five focal planes were taken per wing disc using a 40 × lens. Acquired images were processed using automated Fiji/ImageJ. Generally, Z-stacks were projected, and the channels split. Figures were produced using Adobe photoshop 2022.

### Generation of genetic mosaics

Larvae *yw hs-Flp*^*1.22*^*; MV*-*Dronc-V5-VC/salm-Gal4* were heat shocked as follows in the different experiments:

- for 7 min at 37 °C 48–72 h after egg laying and dissected at the end of third instar larvae (LIII) (Fig. 2a).
- for 30 min at 37 °C 48–72 h after egg laying and dissected at the end of third instar larvae (Supplementary Fig. 3a).

Larvae *w; MV*-*Dronc-V5-VC*/ + ; *dpp-LexA*/*hs-mFlp5* were heat shocked as follows in the different experiments:

- for 60 min at 37 °C 48–72 h after egg laying and dissected at the end of third instar larvae. We noticed that the recombination efficiency using *hs-mFlp5* was lower than that using WT, and a longer heat-shock treatment was needed to generate genetic mosaics.
- Sequentially 2 times for 45 min each at 37 °C 48-72 h after egg laying and dissected at the end of third instar larvae (Supplementary Fig. 3c).

Larvae w; *MV*-*Dronc-V5-VC/hs-Cre; hh-QF* were heat shocked as follows in the different experiments:

- no heat shocked was applied since the *hs-Cre* line has demonstrated leaky expression^7^ (Fig. 2C).
- for 30 min at 37 °C 48-72 h after egg laying and dissected at the end of third instar larvae (Supplementary Fig. 3b).

In all of the experiments the larvae were kept at 25 °C until the end of third instar larvae.

## Supplementary Information

Supplementary Information.

## Data Availability

All data are incorporated into the article and its online supplementary material. All of the experimental resources generated in this manuscript will be publicly available through different public repositories upon publication and until then will be freely distributed upon reasonable request to the corresponding author. The full sequence of the *MV* plasmid is accessible in the Supplementary Text of the manuscript. The plasmids described in the manuscript will be sent to a public repository (e.g., *Drosophila* Genome Resource Repository; https://dgrc.bio.indiana.edu/Home) upon publication. The new *Drosophila* strains described in the manuscript will be submitted to the Bloomington *Drosophila* Stock Center (https://bdsc.indiana.edu/index.html) upon publication.
