# Supplementary material for: A LexAop > UAS > QUAS trimeric plasmid to generate inducible and interconvertible Drosophila overexpression transgenes
Source: Sci Rep. 2022 Mar 9;12:3835. doi: 10.1038/s41598-022-07852-7 (PMC8907290; doi:10.1038/s41598-022-07852-7)
Supplement: Supplementary file 1 — Supplementary Information. [file 41598_2022_7852_MOESM1_ESM.docx]

**
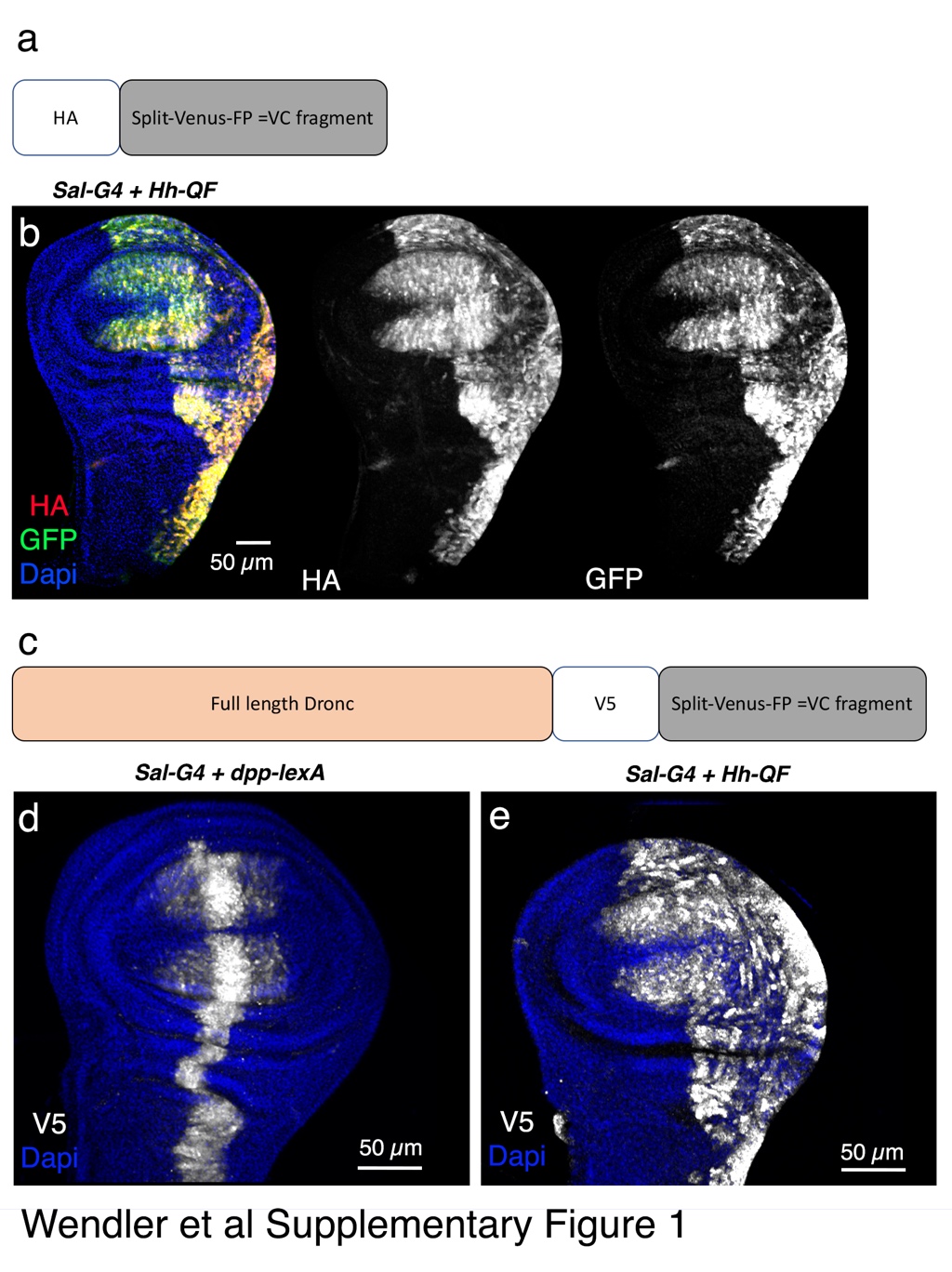
**


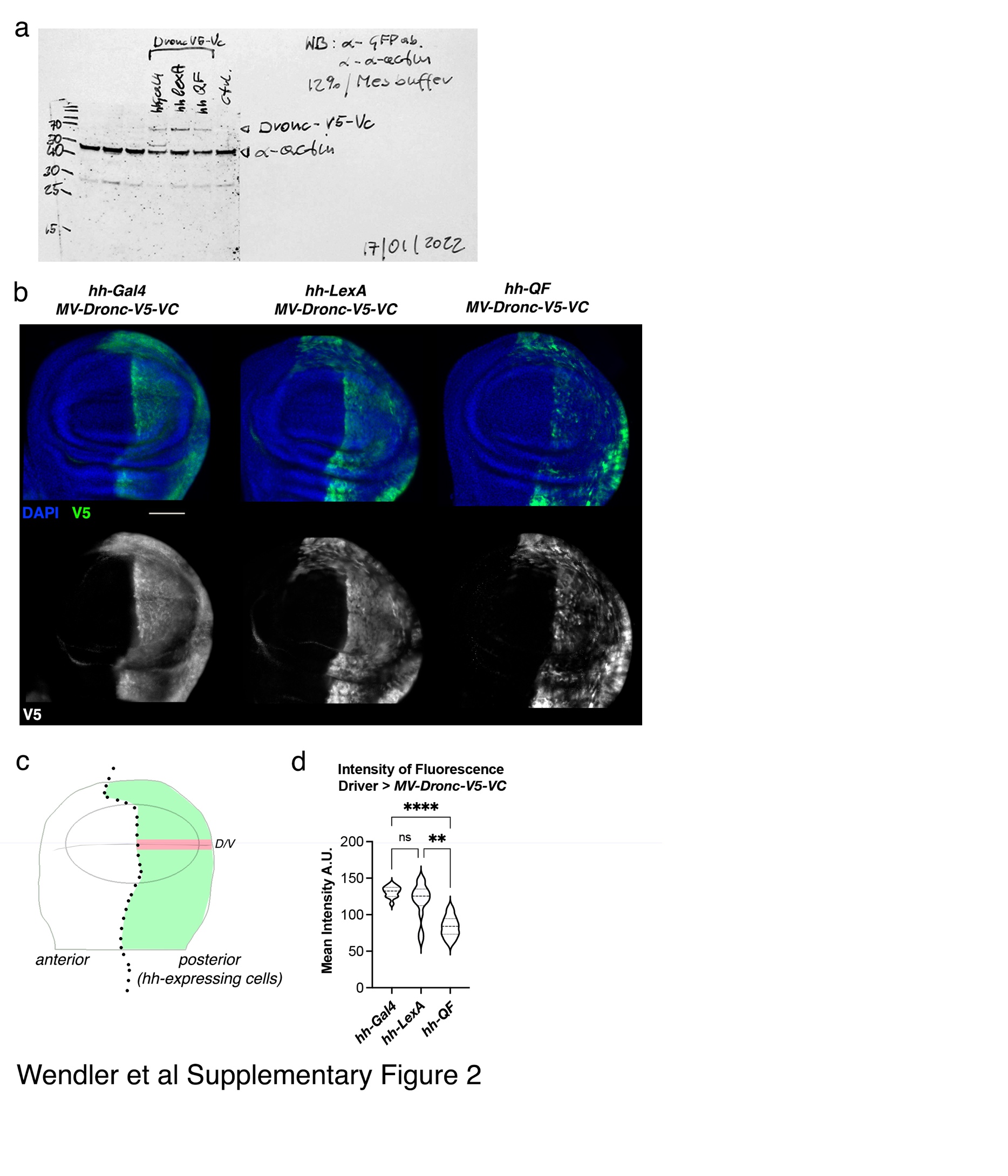


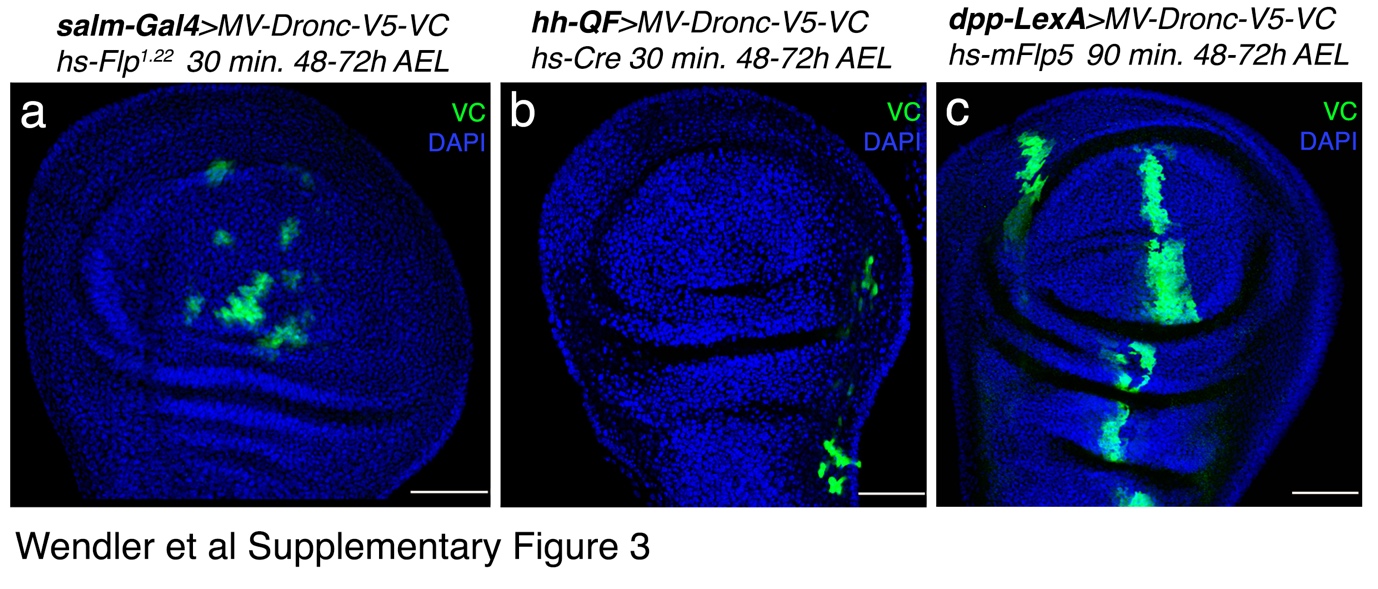


**Supplementary Text.** Whole sequence and main features of ***MV* plasmid**.

> *MV* vector(10,270 bp)

*FRT-71 FRT-WT LoxP* 5x *LexAop* 5x *UAS* 5x *QUAS hsp-70* minimal promoter

Multicloning site *ftz*-intron *SV40-polyA*. *attB* *P-Element* sequences

CATGATGAAATAACATAAGGTGGTCCCGTCGGCAAGAGACATCCACTTAACGTATGCTTGCAATAAGTGCGAGTGAAAGGAATAGTATTCTGAGTGTCGTATTGAGTCTGAGTGAGACAGCGATATGATTGTTGATTAACCCTTAGCATGTCCGTGGGGTTTGAATTAACTCATAATATTAATTAGACGAAATTATTTTTAAAGTTTTATTTTTAATAATTTGCGAGTACGCAAAGCTTGGCTGCATCCAACGCGTGGCGCGCCGAAGTTTCTATACTTTCTAGAGAATAGAAACTGCGGCCGCACTGCTGTATATAAAACCAGTGGTTATATGTACAGTACTGCTGTATATAAAACCAGTGGTTATATGTACAGTACGTCGACTGCTGTATATAAAACCAGTGGTTATATGTACAGTACTGCTGTATATAAAACCAGTGGTTATATGTACAGTACTGCTGTATATAAAACCAGTGGTTATATGTACAGTTAGCGAGCGCCGGAGTATAAATAGAGGCGCTTCGTCTACGGAGCGACAATTCAATTCAAACAAGCAAAGTGAACACGTCGCTAAGCGAAAGCTAAGCAAATAAACAAGCGCAGCTGAACAAGCTAAACAATCTGCAGTAAAGTGCAAGTTAAAGTGAATCAATTAAAAGTAACCAGCAACCAAGTAAATCAACTGCAACTACTGAAATCTGCCAAGAAGTAATTATTGAATACAAGAAGAGAACTCTGAATAGGGAATTGGGGCGGCCGCGAAGTTTCTATACTTTCTAGAGAATAGAAACTGGGGGGGGGGAAGTTCCTATACTTTCTAGAGAATAGGAACTTCGAATTCGCAGGTCGGAGTACTGTCCTCCGAGCGGAGTACTGTCCTCCGAGCGGAGTACTGTCCTCCGAGCGGAGTACTGTCCTCCGAGCGGAGTACTGTCCTCCGAGCGGAGACTCTAGCGAGCGCCGGAGTATAAATAGAGGCGCTTCGTCTACGGAGCGACAATTCAATTCAAACAAGCAAAGTGAACACGTCGCTAAGCGAAAGCTAAGCAAATAAACAAGCGCAGCTGAACAAGCTAAACAATCTGCAGTAAAGTGCAAGTTAAAGTGAATCAATTAAAAGTAACCAGCAACCAAGTAAATCAACTGCAACTACTGAAATCTGCCAAGAAGTAATTATTGAATACAAGAAGAGAACTCTGAATAGGGAATTGGGGCTAGCGAAGTTCCTATACTTTCTAGAGAATAGGAACTTCGGGGGGGGGATAACTTCGTATAATGTATGCTATACGAAGTTATCCTAGGGGGTAATCGCTTATCCTCGGATAAACAATTATCCTCACGGGTAATCGCTTATCCGCTCGGGTAATCGCTTATCCTCGGGTAATCGCTTATCCTTTAGCGAGCGCCGGAGTATAAATAGAGGCGCTTCGTCTACGGAGCGACAATTCAATTCAAACAAGCAAAGTGAACACGTCGCTAAGCGAAAGCTAAGCAAATAAACAAGCGCAGCTGAACAAGCTAAACAATCTGCAGTAAAGTGCAAGTTAAAGTGAATCAATTAAAAGTAACCAGCAACCAAGTAAATCAACTGCAACTACTGAAATCTGCCAAGAAGTAATTATTGAATACAAGAAGAGAACTCTGAATAGGGAATTGGGATAACTTCGTATAATGTATGCTATACGAAGTTATGGGCCCGTTTAAACTCGAGAGGCCTGCGATCGCAGATCTAAGCTTGACGTCGCATGCACCGGTCATATGGGTCACCAGGCCTGGTACGGTACCTCTTGTGAAGGAACCTTACTTCTGTGGTGTGACATAATTGGACAAACTACCTACAGAGATTTAAAGCTCTAAGGTAAATATAAAATTTTTAAGTGTATAATGTGTTAAACTACTGATTCTAATTGTTTGTGTATTTTAGATTCCAACCTATGGAACTGATGAATGGGAGCAGTGGTGGAATGCCTTTAATGAGGAAAACCTGTTTTGCTCAGAAGAAATGCCATCTAGTGATGATGAGGCTACTGCTGACTCTCAACATTCTACTCCTCCAAAAAAGAAGAGAAAGGTAGAAGACCCCAAGGACTTTCCTTCAGAATTGCTAAGTTTTTTGAGTCATGCTGTGTTTAGTAATAGAACTCTTGCTTGCTTTGCTATTTACACCACAAAGGAAAAAGCTGCACTGCTATACAAGAAAATTATGGAAAAATATTTGATGTATAGTGCCTTGACTAGAGATCATAATCAGCCATACCACATTTGTAGAGGTTTTACTTGCTTTAAAAAACCTCCCACACCTCCCCCTGAACCTGAAACATAAAATGAATGCAATTGTTGTTGTTAACTTGTTTATTGCAGCTTATAATGGTTACAAATAAAGCAATAGCATCACAAATTTCACAAATAAAGCATTTTTTTCACTGCATTCTAGTTGTGGTTTGTCCAAACTCATCAATGTATCTTATCATGTCTGGATCCACTAGTGTCGACGATGTAGGTCACGGTCTCGAAGCCGCGGTGCGGGTGCCAGGGCGTGCCCTTGGGCTCCCCGGGCGCGTACTCCACCTCACCCATCTGGTCCATCATGATGAACGGGTCGAGGTGGCGGTAGTTGATCCCGGCGAACGCGCGGCGCACCGGGAAGCCCTCGCCCTCGAAACCGCTGGGCGCGGTGGTCACGGTGAGCACGGGACGTGCGACGGCGTCGGCGGGTGCGGATACGCGGGGCAGCGTCAGCGGGTTCTCGACGGTCACGGCGGGCATGTCGACACTACGCGCCTCGACCTAATTCTAGTATGTATGTAAGTTAATAAAACCCATTTTTGCGGAAAGTAGATAAAAAAAACATTTTTTTTTTTTACTGCACTGGATATCATTGAACTTATCTGATCAGTTTTAAATTTACTTCGATCCAAGGGTATTTGATGTACCAGGTTCTTTCGATTACCTCTCACTCAAAATGACATTCCACTCAAAGTCAGCGCTGTTTGCCTCCTTCTCTGTCCACAGAAATATCGCCGTCTCTTTCGCCGCTGCGTCCGCTATCTCTTTCGCCACCGTTTGTAGCGTTACGTAGCGTCAATGTCCGCCTTCAGTTGCATTTTGTCAGCGGTTTCGTGACGAAGCTCCAAGCGGTTTACGCCATCAATTAAACACAAAGTGCTGTGCCAAAACTCCTCTCGCTTCTTATTTTTGTTTGTTTTTTGAGTGATTGGGGTGGTGATTGGTTTTGGGTGGGTAAGCAGGGGAAAGTGTGAAAAATCCCGGCAATGGGCCAAGAGGATCAGGAGCTATTAATTCGCGGAGGCAGCAAACACCCATCTGCCGAGCATCTGAACAATGTGAGTAGTACATGTGCATACATCTTAAGTTCACTTGATCTATAGGAACTGCGATTGCAACATCAAATTGTCTGCGGCGTGAGAACTGCGACCCACAAAAATCCCAAACCGCAATTGCACAAACAAATAGTGACACGAAACAGATTATTCTGGTAGCTGTTCTCGCTATATAAGACAATTTTTGAGATCATATCATGATCAAGACATCTAAAGGCATTCATTTTCGACTATATTCTTTTTTACAAAAAATATAACAACCAGATATTTTAAGCTGATCCTAGATGCACAAAAAATAAATAAAAGTATAAACCTACTTCGTAGGATACTTCGGGGTACTTTTTGTTCGGGGTTAGATGAGCATAACGCTTGTAGTTGATATTTGAGATCCCCTATCATTGCAGGGTGACAGCGGAGCGGCTTCGCAGAGCTGCATTAACCAGGGCTTCGGGCAGGCCAAAAACTACGGCACGCTCCGGCCACCCAGTCCGCCGGAGGACTCCGGTTCAGGGAGCGGCCAACTAGCCGAGAACCTCACCTATGCCTGGCACAATATGGACATCTTTGGGGCGGTCAATCAGCCGGGCTCCGGATGGCGGCAGCTGGTCAACCGGACACGCGGACTATTCTGCAACGAGCGACACATACCGGCGCCCAGGAAACATTTGCTCAAGAACGGTGAGTTTCTATTCGCAGTCGGCTGATCTGTGTGAAATCTTAATAAAGGGTCCAATTACCAATTTGAAACTCAGTTTGCGGCGTGGCCTATCCGGGCGAACTTTTGGCCGTGATGGGCAGTTCCGGTGCCGGAAAGACGACCCTGCTGAATGCCCTTGCCTTTCGATCGCCGCAGGGCATCCAAGTATCGCCATCCGGGATGCGACTGCTCAATGGCCAACCTGTGGACGCCAAGGAGATGCAGGCCAGGTGCGCCTATGTCCAGCAGGATGACCTCTTTATCGGCTCCCTAACGGCCAGGGAACACCTGATTTTCCAAGCCATGGTGCGGATGCCACGACATCTGACCTATCGGCAGCGAGTGGCCCGCGTGGATCAGGTGATCCAGGAGCTTTCGCTCAGCAAATGTCAGCACACGATCATCGGTGTGCCCGGCAGGGTGAAAGGTCTGTCCGGCGGAGAAAGGAAGCGTCTGGCATTCGCCTCCGAGGCTCTAACCGATCCGCCGCTTCTGATCTGCGATGAGCCCACCTCCGGACTGGACTCCTTTACCGCCCACAGCGTCGTCCAGGTGCTGAAGAAGCTGTCGCAGAAGGGCAAGACCGTCATCCTGACCATTCATCAGCCGTCTTCCGAGCTGTTTGAGCTCTTTGACAAGATCCTTCTGATGGCCGAGGGCAGGGTAGCTTTCTTGGGCACTCCCAGCGAAGCCGTCGACTTCTTTTCCTAGTGAGTTCGATGTGTTTATTAAGGGTATCTAGTATTACATAACATCTCAACTCCTATCCAGCGTGGGTGCCCAGTGTCCTACCAACTACAATCCGGCGGACTTTTACGTACAGGTGTTGGCCGTTGTGCCCGGACGGGAGATCGAGTCCCGTGATCGGATCGCCAAGATATGCGACAATTTTGCCATTAGCAAAGTAGCCCGGGATATGGAGCAGTTGTTGGCCACCAAAAATCTGGAGAAGCCACTGGAGCAGCCGGAGAATGGGTACACCTACAAGGCCACCTGGTTCATGCAGTTCCGGGCGGTCCTGTGGCGATCCTGGCTGTCGGTGCTCAAGGAACCACTCCTCGTAAAAGTGCGACTTATTCAGACAACGGTGAGTGGTTCCAGTGGAAACAAATGATATAACGCTTACAATTCTTGGAAACAAATTCGCTAGATTTTAGATAGAATTGCCTGATTCCACACCCTTCTTAGTTTTTTTCAATGAGATGTATAGTTTATAGTTTTGCAGAAGATAAATAAATTTCATTTAACTCGCGAATATTAATGAGATGCGAGTAACATTTTAATTTGCAGATGGTTGCCATCTTGATTGGCCTCATCTTTTTGGGCCAACAACTCACGCAAGTGGGTGTGATGAATATCAACGGAGCCATCTTCCTCTTCCTGACCAACATGACCTTTCAAAACGTCTTTGCCACGATAAATGTAAGTCATGTTTAGAATACATTTGCATTTCAATAATTTACTAACTTTCTAATGAATCGATTCGATTTAGGTGTTCACCTCAGAGCTGCCAGTTTTTATGAGGGAGGCCCGAAGTCGACTTTATCGCTGTGACACATACTTTCTGGGCAAAACGATTGCCGAATTGCCGCTTTTTCTCACAGTGCCACTGGTCTTCACGGCGATTGCCTATCCGATGATCGGACTGCGGGCCGGAGTGCTGCACTTCTTCAACTGCCTGGCGCTGGTCACTCTGGTGGCCAATGTGTCAACGTCCTTCGGATATCTAATATCCTGCGCCAGCTCCTCGACCTCGATGGCGCTGTCTGTGGGTCCGCCGGTTATCATACCATTCCTGCTCTTTGGCGGCTTCTTCTTGAACTCGGGCTCGGTGCCAGTATACCTCAAATGGTTGTCGTACCTCTCATGGTTCCGTTACGCCAACGAGGGTCTGCTGATTAACCAATGGGCGGACGTGGAGCCGGGCGAAATTAGCTGCACATCGTCGAACACCACGTGCCCCAGTTCGGGCAAGGTCATCCTGGAGACGCTTAACTTCTCCGCCGCCGATCTGCCGCTGGACTACGTGGGTCTGGCCATTCTCATCGTGAGCTTCCGGGTGCTCGCATATCTGGCTCTAAGACTTCGGGCCCGACGCAAGGAGTAGCCGACATATATCCGAAATAACTGCTTGTTTTTTTTTTTTACCATTATTACCATCGTGTTTACTGTTTATTGCCCCCTCAAAAAGCTAATGTAATTATATTTGTGCCAATAAAAACAAGATATGACCTATAGAATACAAGTATTTCCCCTTCGAACATCCCCACAAGTAGACTTTGGATTTGTCTTCTAACCAAAAGACTTACACACCTGCATACCTTACATCAAAAACTCGTTTATCGCTACATAAAACACCGGGATATATTTTTTATATACATACTTTTCAAATCGCGCGCCCTCTTCATAATTCACCTCCACCACACCACGTTTCGTAGTTGCTCTTTCGCTGTCTCCCACCCGCTCTCCGCAACACATTCACCTTTTGTTCGACGACCTTGGAGCGACTGTCGTTAGTTCCGCGCGATTCGGTTCGCTCAAATGGTTCCGAGTGGTTCATTTCGTCTCAATAGAAATTAGTAATAAATATTTGTATGTACAATTTATTTGCTCCAATATATTTGTATATATTTCCCTCACAGCTATATTTATTCTAATTTAATATTATGACTTTTTAAGGTAATTTTTTGTGACCTGTTCGGAGTGATTAGCGTTACAATTTGAACTGAAAGTGACATCCAGTGTTTGTTCCTTGTGTAGATGCATCTCAAAAAAATGGTGGGCATAATAGTGTTGTTTATATATATCAAAAATAACAACTATAATAATAAGAATACATTTAATTTAGAAAATGCTTGGATTTCACTGGAACTAGAATTAATTCGGCTGCTGCTCTAAACGACGCATTTCGTACTCCAAAGTACGAATTTTTTCCCTCAAGCTCTTATTTTCATTAAACAATGAACAGGACCTAACGCACAGTCACGTTATTGTTTACATAAATGATTTTTTTTACTATTCAAACTTACTCTGTTTGTGTACTCCCACTGGTATAGCCTTCTTTTATCTTTTCTGGTTCAGGCTCTATCACTTTACTAGGTACGGCATCTGCGTTGAGTCGCCTCCTTTTAAATGTCTGACCTTTTGCAGGTGCAGCCTTCCACTGCGAATCATTAAAGTGGGTATCACAAATTTGGGAGTTTTCACCAAGGCTGCACCCAAGGCTCTGCTCCCACAATTTTCTCTTAATAGCACACTTCGGCACGTGAATTAATTTTACTCCAGTCACAGCTTTGCAGCAAAATTTGCAATATTTCATTTTTTTTTATTCCACGTAAGGGTTAATGTTTTCAAAAAAAAATTCGTCCGCACACAACCTTTCCTCTCAACAAGCAAACGTGCACTGAATTTAAGTGTATACTTCGGTAAGCTTCGGCTATCGACGGGACCACCTTATGTTATTTCATCATGGGCCAGACCCACGTAGTCCAGCGGCAGATCGGCGGCGGAGAAGTTAAGCGTCTCCAGGATGACCTTGCCCGAACTGGGGCACGTGGTGTTCGACGATGTGCAGCTAATTTCGCCCGGCTCCACGTCCGCCCATTGGTTAATCAGCAGACCCTCGTTGGCGTAACGGAACCATGAGAGGTACGACAACCATTTGAGGTATACTGGCACCGAGCCCGAGTTCAAGAAGAAGGCGTTTTTCCATAGGCTCCGCCCCCCTGACGAGCATCACAAAAATCGACGCTCAAGTCAGAGGTGGCGAAACCCGACAGGACTATAAAGATACCAGGCGTTTCCCCCTGGAAGCTCCCTCGTGCGCTCTCCTGTTCCGACCCTGCCGCTTACCGGATACCTGTCCGCCTTTCTCCCTTCGGGAAGCGTGGCGCTTTCTCATAGCTCACGCTGTAGGTATCTCAGTTCGGTGTAGGTCGTTCGCTCCAAGCTGGGCTGTGTGCACGAACCCCCCGTTCAGCCCGACCGCTGCGCCTTATCCGGTAACTATCGTCTTGAGTCCAACCCGGTAAGACACGACTTATCGCCACTGGCAGCAGCCACTGGTAACAGGATTAGCAGAGCGAGGTATGTAGGCGGTGCTACAGAGTTCTTGAAGTGGTGGCCTAACTACGGCTACACTAGAAGAACAGTATTTGGTATCTGCGCTCTGCTGAAGCCAGTTACCTTCGGAAAAAGAGTTGGTAGCTCTTGATCCGGCAAACAAACCACCGCTGGTAGCGGTGGTTTTTTTGTTTGCAAGCAGCAGATTACGCGCAGAAAAAAAGGATCTCAAGAAGATCCTTTGATCTTTTCTACGGGGTCTGACGCTCAGTGGAACGAAAACTCACGTTAAGGGATTTTGGTCATGAGATTATCAAAAAGGATCTTCACCTAGATCCTTTTAAATTAAAAATGAAGTTTTAAATCAATCTAAAGTATATATGAGTAAACTTGGTCTGACAGTTACCAATGCTTAATCAGTGAGGCACCTATCTCAGCGATCTGTCTATTTCGTTCATCCATAGTTGCCTGACTCCCCGTCGTGTAGATAACTACGATACGGGAGGGCTTACCATCTGGCCCCAGTGCTGCAATGATACCGCGAGACCCACGCTCACCGGCTCCAGATTTATCAGCAATAAACCAGCCAGCCGGAAGGGCCGAGCGCAGAAGTGGTCCTGCAACTTTATCCGCCTCCATCCAGTCTATTAATTGTTGCCGGGAAGCTAGAGTAAGTAGTTCGCCAGTTAATAGTTTGCGCAACGTTGTTGCCATTGCTACAGGCATCGTGGTGTCACGCTCGTCGTTTGGTATGGCTTCATTCAGCTCCGGTTCCCAACGATCAAGGCGAGTTACATGATCCCCCATGTTGTGCAAAAAAGCGGTTAGCTCCTTCGGTCCTCCGATCGTTGTCAGAAGTAAGTTGGCCGCAGTGTTATCACTCATGGTTATGGCAGCACTGCATAATTCTCTTACTGTCATGCCATCCGTAAGATGCTTTTCTGTGACTGGTGAGTACTCAACCAAGTCATTCTGAGAATAGTGTATGCGGCGACCGAGTTGCTCTTGCCCGGCGTCAATACGGGATAATACCGCGCCACATAGCAGAACTTTAAAAGTGCTCATCATTGGAAAACGTTCTTCGGGGCGAAAACTCTCAAGGATCTTACCGCTGTTGAGATCCAGTTCGATGTAACCCACTCGTGCACCCAACTGATCTTCAGCATCTTTTACTTTCACCAGCGTTTCTGGGTGAGCAAAAACAGGAAGGCAAAATGCCGCAAAAAAGGGAATAAGGGCGACACGGAAATGTTGAATACTCATACTCTTCCTTTTTCAATATTATTGAAGCATTTATCAGGGTTATTGTCTCATGAGCGGATACATATTTGAATGTATTTAGAAAAATAAACAAATAGGGGTTCCGCGCACATTTCCCCGAAAAGTGCCACCTGACGTCTAAGAAACCATTATTATCATGACATTAACCTATAAAAATAGGCGTATCACGAGGCCCTTTCGTCTCGCGCGTTTCGGTGATGACGGTGAAAACCTCTGACACATGCAGCTCCCGGAGACGGTCACAGCTTGTCTGTAAGCGGATGCCGGGAGCAGACAAGCCCGTCAGGGCGCGTCAGCGGGTGTTGGCGGGTGTCGGGGCTGGCTTAACTATGCGGCATCAGAGCAGATTGTACTGAGAGTGCACCATATGCGGTGTGAAATACCGCACCGAATCGCGCGGAACTAACGACAGTCGCTCCAAGGTCGTCGAACAAAAGGTGAATGTGTTGCGGAGAGCGGGTGGGAGACAGCGAAAGAGCAACTACGAAACGTGGTGTGGTGGAGGTGAATTATGAAGAGGGCGCGCGATTTGAAAAGTATGTATATAAAAAATATATCCCGGTGTTTTATGTAGCGATAAACGAGTTTTTGATGTAAGGTATGCAGGTGTGTAAGTCTTTTGGTTAGAAGACAAATCCAAAGTCTACTTGTGGGGATGTTCGAAGGGGAAATACTTGTATTCTATAGGTCATATCTTGTTTTTATTGGCACAAATATAATTACATTAGCTTTTTGAGGGGGCAATAAACAGTAAACACGATGGTAATAATGGTAAAAAAAAAAAACAAGCAGTTATTTCGGATATATGTCGGCTACTCCTTGCGTCGGGCCCGAAGTCTTAGAGCCAGATATGCGAGCACCCGGAAGCTCACGATGAGAATGGCCAGAC

**Supplementary Figure 1**

**a)** Schematic showing the design of the *HA-VC* construct. **b**) Concomitant expression of the HA-VC protein in the *salm-Gal4* and *hh-QF* cellular domains of the wing disc; the HA-VC expression was detected by using anti-HA (red) and anti-GFP (green) antibodies. **c)** Schematic showing the design of the *Dronc-V5-VC* construct. **d-e)** Concomitant expression of the Dronc-V5-VC (grey anti-V5) in several territories of the wing disc induced by different transcriptional activators. DAPI stains DNA in the entire figure (blue). Scale bars represent 50 μm in the entire figure.

**Supplementary Figure 2**

**a)** Raw data of the picture shown in Figure 1g. Western Blot shows the expression of the full-length Dronc-V5-VC construct induced by *hh-Gal4, hh-LexA*, and *hh-QF*. The control empty (CTRL) sample was obtained from flies *hh-lexA* without the *MV-Dronc-V5-VC* transgene. The expected sizes of Dronc-V5-VC and Actin are approximately 62 and 42 kDa, respectively. **b)** Representative images of wing imaginal discs expressing Dronc-V5-VC (anti V5 in green) under the regulation of *hh-Gal4, hh-LexA,* and *hh-QF*. DAPI labels DNA (blue). Scale bar: 50μm. **c)** Schematic showing a wing disc in which the posterior compartment is coloured in green; we estimated the fluorescence intensity of V5 immunostaining in d within the indicated region in red. The mean intensity of fluorescence was estimated by tracing 50 parallel straight lines (1 point spaced) around the Dorso-Ventral boundary (D/V) using Fiji and the corresponding measuring plugin. **d)** Mean fluorescence intensity (A.U.) of V5 immunostaining in the P compartment of wing discs of the genotypes indicated in b. Statistical significance was determined using one way ANOVA with Dunn’s multiple comparison; not significant, n.s., ** p<0.01, ****p<0.0001. number of discs *hh-Gal4* n = 14; *hh-lexA* n = 14; *hh-QF* n = 20. N=2. Full genotype descriptions of the figure can be found in Methods.

**Supplementary Figure 3**

**a-c)** Clonal elimination from the genome of 5X *UAS* (a), 5X *QUAS* (b), and 5X *LexAop* (c) binding sites upon random and prolonged exposure to Flp^1.22^ (a), Cre (b) and mFlp5 (c); anti-GFP antibody was used to detect the VC tag of the Dronc-V5-VC construct (green). DAPI (blue) labels the DNA. Notice the large elimination of the VC immunoreactivity in the *salm-* and *hh*-expressing cells as opposed to the persistence in the *dpp-LexA* expressing cells. Compare the expression of Dronc-V5-VC (green) in this figure with the Supplementary Fig. 1d and 1e. Scale bars represent 50 μm in the entire figure.
